# The costs of traumatic head injury and associated factors at University of Gondar Specialized Referral Hospital, Northwest Ethiopia

**DOI:** 10.1186/s12889-019-7800-3

**Published:** 2019-10-28

**Authors:** Yilak Asmamaw, Mezgebu Yitayal, Ayal Debie, Simegnew Handebo

**Affiliations:** 1 Medical Ward, University of Gondar Specialized Referral Hospital, Gondar, Ethiopia; 2 Department of Health Systems and Policy, Institute of Public Health, University of Gondar, Gondar, Ethiopia; 3 Department of Health Education and Behavioral Sciences, Institute of Public Health, University of Gondar, Gondar, Ethiopia

**Keywords:** Cost, Traumatic head injury, Gondar, Ethiopia

## Abstract

**Background:**

Head injuries account for 650,000 annual deaths worldwide. The cost for treating head injury was estimated at US $200 million annually. This contributes to economic impoverishment in low income countries like Ethiopia. Hence, this study was aimed to assess the cost of Traumatic Head Injury (THI) and associated factors in the University of Gondar Specialized Referral Hospital.

**Method:**

An institution-based cross-sectional study was conducted from March 01 to May 30, 2017. A total of 387 THI patients were included in the study. An interviewer-administered questionnaire was used for data collection. Direct costs and indirect costs were measured by using the bottom-up approach. Data were entered into Epi-Info version 7 and imported to SPSS version 20 for analysis. Simple and multiple linear regression analysis were done to identify factors associated with cost of THI.

**Results:**

The mean cost of THI per patient was 4673.43 Ethiopian Birr (ETB), 95% CI (4523.6-4823.3), and length of hospital stay averaged 1.73, 95% CI (1.63–1.82). Direct non-medical cost, like transportation fee 1896.19 ETB (±762.56 SD) and medical costs 1101.66 ETB (±534.13 SD) were account for 40.57 and 23.58% of total costs respectively. The indirect cost, loss of income by patient and their attendant due to injury, was 1675.58 ETB (+ 459.26 SD). Patients with moderate and severe levels of injury have 635.167 ETB (Standardized coefficient = 0.173, *p* < 0.001) and 773.621 ETB (Standardized coefficient = 0. 132, p < 0.001) increased costs, respectively, compared to mild level THI patients. Costs for patients ages 31–45 years were 252.504 ETB (Standardized coefficient = − 0.066, *p* = 0.046) lower than costs for those 5–14 years old. The cost of THI patients increased by 1022.853 ETB for each additional day of hospital length of stay (Standardized coefficient = 0.648, *p* < 0.001).

**Conclusion:**

Most expenses of the THI were from direct non-medical cost. Prior health service use, length of stay, level of injury, and age were significant predictors of cost of THI.

## Background

Injuries are a critical public health problem worldwide which accounts for 1 of the 5 major causes of death and disability in the world. It leads to about 5 million deaths per year, which mean 16,000 deaths daily [1, 2]. A THI is defined as any injury to the head caused by physical damage/structural change to the scalp or skull due to any type of external force with or without other body organ injury. Most common causes are falls, road accidents, collisions and violence [3].

THI will be the major cause of death and disability by the year 2020. It has been estimated that THI affects over 10 million people annually [4]. This makes THI a pressing public health and medical problem. The current evidences indicate that nearly 60% of THIs are due to road traffic injuries in all parts of the world [5, 6].

THI mostly occurs among people between the ages of 15 and 45 years, those believed to be the most productive members of the community. It causes substantial morbidity, disability, and mortality, and is associated with high health care costs, both for individuals and society. Additionally, members of a household might lose many productive days of their lives. This loss, when added to the costs of treatment, might push the poor household further into poverty, and it has a great impact on growth and economic development of a nation [7–10]. The average costs of THI vary significantly by age, Glasgow Coma Score (GCS), Injury Severity Score, co-existing injuries of the thorax, spine and lower limb, hospital mortality, and availability of neurosurgical services [11, 12].

One New Zealand study showed that 95% THI patients had mild injury, of which approximately 64% were not initially hospitalized. Of those who were hospitalized, survivors spent on average 2.6 days (95% CI 1.8–3.3) in the hospital. The total direct health care cost over 12 months was US $3783 per patient (minimum US $26, maximum US $112,115). In addition, costs attributable to production losses were estimated US$2000 (minimum US$163, maximum US $13,252) [7].

A recent review on THI showed that in US head injury accounts for 13% of all hospitalized injuries, 70% of which were minor head injuries. The annual estimated cost of all head injuries was US$200 million [12, 13]. Severity of injury (mild, moderate, and sever), length of hospital stay and age of participants were significantly associated with cost [12, 14].

Direct out-of-pocket payments incurred by households for medical services received are estimated to account for 23% of total global health expenditure and 45% of health expenditure in the developing world [15]. The Ethiopian health system is highly dependent on out-of-pocket payments by households [16, 17]. In the country, 59 and 88% of those who sought outpatient and inpatient care covered through out-of-pocket payments, respectively. The out-of-pocket payments by households contribute to about 34% of the total health expenditure in Ethiopia [18].

In order to assist health service planning, understanding factors that influence the costs (typically out-of pocket payment) is essential. THI is one of the major causes of hospitalization, disability and death in Ethiopia. Hence, the information from this study is valuable in policy, planning, and decision making related to the prevention of THI and managing the associated cost of health care services. Therefore, this study will analyze the direct out-of-pocket and indirect cost of THI at the ED of University of Gondar Specialized Hospital, Northwest Ethiopia.

## Methods

### Study area and design

An institution-based cross-sectional study was conducted at the University of Gondar Specialized Referral Hospital from March to May, 2017. It is one of the pioneer teaching hospital in the country. It is also a referral site for many health centers and hospitals in Northwest Ethiopia. The hospital is located in the city of Gondar in Northwest Ethiopia, nearly 727 km from the capital city of Addis Ababa. The hospital provides services for a population of more than 5 million people.

### Study population and sample size

All THI patients treated at University of Gondar Specialized Hospital ED during the study period were included in the study. However, patients with neurological diseases before the traumatic head injury, brain injury with non-traumatic causes, or vegetative state (severe loss of consciousness) were excluded from the study. These health conditions over the cost due to previous health problem rather than merely THI. The sample size was calculated using the single population proportion formula considering the following assumptions: Proportion of traumatic head injury patients incurred above the mean cost of injury was taken to be 50, 95% level of confidence, 0.05 margin of error and 5% non-response rate the final sample size was 403. The average monthly traumatic head injury patient flow of the preceding year and the month prior to the actual data collection period was estimated from ED registration book. As a result, due to low THI patient flow all patients visited the hospital with THI case during the study period were involved in the study [19].

### Study variables

#### Head injury

Defined as any injury to the brain caused by physical damage / structural change to the scalp or skull due to any type of external force to the head.

#### Cost of traumatic head injury

the sum of all out-of-pocket payments incurred by the patient or his family for different expenses related to the injury as well as loss of production due to the injury.

#### Direct costs

the sum of expenses of medical care in hospitals, transportation, laboratory tests, clinical procedures, and food and services incurred because of the injury.

#### Direct medical costs

the sum of all costs for consultation fee, cost of medicines, costs of investigation in the hospital.

#### Direct non-medical costs

the sum of all costs for transportation, food, and accommodation.

#### Indirect costs

represent the value of lost production because of reduced working time or impaired performance at the workplace.

#### Severity level

the Glasgow Coma Scale (GCS) defines the scores of 13–15, 9–12 and 3–8 as mild, moderate, and severe head injury, respectively [20].

#### ED Length of stay

the median time from arrival to ED departure for admitted patients and for discharged patients [21].

### Data collection procedures

The patients and/or their care givers were approached for the interview in the ED after they got all necessary treatments. The questionnaire included questions about socio-demographics, wealth index, direct cost of THI, indirect cost of THI and severity level of THI. The wealth index variable was derived from the different assets of the households to assess the household cumulative wealth status. In the dataset, the wealth index were presented as “Poor”, “Middle” and “high” categories. A structured, interviewer-administered questionnaire was used to collect the data. The questionnaire was also pretested in Gondar Poly health center ED with similar case patients. The direct out of pocket expenses (in ETB) for care were recorded, including hospitalization, laboratory tests, medication, transportation, and surgical procedures. The indirect cost was measured by productivity losses due to morbidity and mortality, borne by the individual and/or family members. Two supervisors and eight data collectors participated in data collection, each of whom received a one-day training by the principal investigator.

### Data processing and analysis

After coding, the data were entered into EPI INFO 7 and exported to Statistical Package for Social Science (SPSS) version 20 for analysis. Descriptive statistics were computed using standard statistical parameters. After normality and homoscedasticity assumptions were checked (the outcome variable was normally distributed), simple univariate linear regression analysis was done to assess the association between each independent variable and the cost of illness. Finally, independent variables with *p*-values less than 0.2 were included in the final multiple linear regression model. Un-standardized coefficients were used to interpret effects on the dependent variable. Covariates were deemed significant if p-values were less than 0.05.

## Results

### Socio-demographic characteristics

Out of 403 THI patients 387 respondents participated in the study, resulting in a response rate of 96.03%. Nine (2.23%) people refused to participate and 7 (1.74%) cases were excluded because of their expenditure on severe unrelated diseases. As presented in Table 1, the majority, 301(77.78%), were males. One hundred fifty five (40.1%) were farmers. One hundred thirty five (34.6%) had a primary level education. Nearly half of the participants were age 15–30, and 193 (49.9%) were married. The mean family size was 4.48 ± (1.92 SD) persons per household. The mean monthly income was 3901 ± (2503.59 ETB SD).

**Table 1:** Socio demographic characteristics of patients with traumatic head injury at University of Gondar Specialized Referral Hospital, Northwest Ethiopia (*n* = 387)

Characteristics		Frequency	Percent (%)
Sex	Male	301	77.78
	Female	86	22.22
Age (years)	5–14	60	15.5
	15–30	197	50.9
	31–45	73	18.9
	≥46	57	14.7
Occupation of respondents	Currently not working	44	11.4
	Civil servant	85	22.0
	Daily labor	103	26.6
	Farmer	155	40.1
Marital status	Single	189	48.8
	Married	193	49.9
	Divorced	2	0.5
	Widowed	3	0.8
Educational status	Unable to read & write	68	17.6
	Able to read & write	95	24.5
	Primary level	134	34.6
	Secondary & above	90	23.3
Family size of respondent	1–3	116	30.0
	4–5	156	40.3
	6 and above	115	29.7

### Injury characteristics

Out of the study participant 101 (26.1%) had visited other health institution prior to Gondar University Specialized hospital. The majority 192 (49.5%), of THIs were caused by motor vehicle accidents, followed by violence 157 (40.56%). Most of the accidents occurred between noon and midnight. Based on the GCS, 281 (72.6%) of participants had mild THI, 79 (20.41%) experienced moderate injuries, and 27 (6.98%) were severe. Of the total subjects, 300 (77.5%) were admitted to inpatient, 80 (20.7%) were discharged from the ED within 24 h, and 7 (1.81%) patients died in the ED.

### Cost of traumatic head injury

The mean cost per patient was 4673.21 ETB (±1499.24 SD) with an average length of stay 1.73 days, 95% CI (1.63–1.82). This mean incurred cost was different across mild, moderate and severe level of injuries (*p* = 0.001). Direct non-medical cost 1896.19 ETB (±762.56 SD) and medical costs 1101.66 ETB (±534.13 SD) account for 40.57 and 23.58% of total costs respectively Table 2. Three hundred frothy three (88.6%) respondents reported lost working days and income associated with their illness. The mean lost income for the attendants due to the stay at the ED was 398.23 ETB (±70.06 SD). Each patient had at least three family member or relatives assisting them. The mean loss of working days of the attendant was 7.68 (±1.06 SD) with the mean loss of income of 1277.35 ETB (±389.20 SD). Total indirect cost for the episode of THI for the patient and their relative(s) was 1675.565 ETB (± 459.26 SD) Table 3.

**Table 2:** Direct costs of trauma head injury patients at University of Gondar Specialized Referral Hospital Northwest, Ethiopia (*n* = 387)

Variable		Before reaching the hospital		At Hospital	
		Mean	Standard Deviation	Mean	Standard Deviation
Direct medical cost	Card registration fee	2**.**52	.30	5.53	1.420
	Investigation & procedure fee	14.90	1.36	816.95	328.05
	Drug and emergency service fee	34.5	10.15	227.26	192.85
Direct non-medical cost	Transportation fee	38.10	11.27	1782.85	729.08
	Other expenses	10.20	2.18	65.04	20.03
Total direct costs ^*^		100.22	25.26	2897.63	1271.43

^*^ Cost = ETB

**Table 3:** Indirect costs of head injury patients and attendants at University of Gondar Specialized Referral Hospital Northwest, Ethiopia (n = 387)

Variables	Mean	Standard Deviation
Length of stay at ED	1.73	0.95
Number of attendants per patient	3	2.56
Workdays lost by attendants	7.68	1.06
Workdays lost patients	2.61	1.16
Loss of income of patients^*^	398.23	70.06
Loss of income of attendants^*^	1277.35	389.20

^*^ Cost: ETB

### Factors associated with cost of head injury

In simple linear regression analysis sex, age, service used before arrival at ED, length of stay at ED, educational status, level of injury, type of trauma, wealth index, and family size were significantly associated with the cost of THI in the ED. The multivariate regression model was statistically significant (F _[_8_]_, 386 = 41.8, *p* < 0.000.), and had an adjusted R square = .615. Factors significantly associated with cost of THI include: previous use of health services at other health institutions, length of stay at ED, level of injury, and age. Patients with moderate and severe levels of injury had 635.167 ETB (Standardized coefficient = 0.173, *p* < 0.001) and 773.621 ETB (Standardized coefficient = 0. 132, *p* < 0.001) higher costs, respectively, compared to patients with mild THI. Patients aged 31–45 had 252.504 ETB (Standardized coefficient = − 0.066, *p* = 0.046) lower costs compared to patients in the 5–14 age group. The costs of THI patients increased 1022.853 ETB for each additional day of hospital stay (Standardized coefficient = 0.648, p < 0.001) Table 4.

**Table 4:** Factors associated with the cost of traumatic head injury at University of Gondar Specialized Referral Hospital Northwest, Ethiopia (n = 387)

Variables	Un-standardized Coefficients		Standardized Coefficients	Sig.	95.0% Confidence Interval for B	
	B	Std. Error	Beta		Lower Bound	Upper Bound
(Constant)	3190.897	288.326		.000	2623.953	3757.841
Sex of respondents	−69.209	119.243	−.019	.562	−303.679	165.261
Age 31–45	−252.504	126.119	−.066	.046	− 500.494	−4.513
Family size 4–5	178.577	115.604	.059	.123	−48.738	405.893
Family size > = 6	66.004	129.415	.020	.610	− 188.469	320.477
High wealth index	243.751	143.618	.064	.090	−38.648	526.150
Able to write and read	−4.812	123.414	−.001	.969	− 247.484	237.860
Primary education	− 113.347	114.067	−.036	.321	− 337.641	110.947
Service used before Hospital	−231.149	117.681	−.070	.050	− 462.548	.249
Length of stay at Hospital	1022.853	58.965	.648	.000	906.909	1138.797
Moderate injury level	635.167	120.675	.173	.000	397.881	872.453
Severe injury level	773.621	205.557	.132	.000	369.429	1177.813
Motor bicycle accident	−81.411	96.620	−.027	.400	−271.398	108.575

Cost- ETB; Reference group: Male, 5–14 age group, poor wealth index, secondary and above, service used and mild THI

## Discussion

This study investigates the cost of THI among patients attended by the University of Gondar Specialized hospital ED. THI affects mostly productive age groups. It causes substantial morbidity, disability, mortality and high health care costs, both for individuals and society [9, 10]. In this study the mean cost of THI per patient at the ED was 4673.43 ETB, 95% CI (4523.6–4823.3), with an average length of stay of 1.73 days, 95% CI (1.63–1.82). Direct non-medical costs and direct medical costs account for 40.6 and 23.6% of total costs, respectively.

This study found that THI accounts for 33.2% of ED visits at the University of Gondar Specialized Hospital. This is higher than shown in the literature for other countries [12, 22]. This may be due to higher prevalence of THI in low and middle income counties than in developed nations. Nearly half (49.5%) of head injuries in Gondar were caused by motor vehicle accidents. This differs from studies conducted in the United States and Australia in which most causes of THIs were related to falls [23, 24].

Using the GCS, we found that 72.6% of the participants had mild THIs. This finding is similar to most previous studies, Mild THI accounts for 70 to 90% of all THI cases [23, 25]. However, it is lower than the proportion found in one New Zealand study in which nearly 95% of TBI cases were considered mild [7]. This may be due to the difference in the causes of THI; in this study almost half the THI cases were a result of motor accidents. Of the study participants, 77.5% were admitted to the hospital inpatient department and only 1.81% died in the emergency room. This finding is lower than a study conducted in England and Wales [12]. This difference may be due to differences in health care service between the study areas.

This study revealed the mean cost of THI per patient seen at the ED was 4673.43 ETB, 95% CI (4523.6–4823.3), and the mean hospital length of stay was 1.73 days. This is lower than the findings of many other studies done in developing and developed countries [7, 12, 26, 27]. This difference may be due to differences in socioeconomic factors and health care price. Direct non-medical cost accounted for 40.57% of the total costs, and 61.5% of this cost was for transportation services. This study’s findings were different from studies done in developed countries where the majority of the costs were direct medical costs [7, 12, 26, 27]. This may be due to most injuries in the Gondar area occurring late at night when there is insufficient transport service availability. With regard to indirect costs, the mean loss of working days of the caregiver (typically a family member) was 7.8, with the loss of income averaging 1277.07 ETB (±1106.695SD).

In this study, the cost of THI was most affected by: the patient’s previous use of another healthcare organization’s services before coming to the ED, length of stay, level of injury, and age. The cost of THI significantly increases with the level of injuries. Moderate and severe THI patients cost 635.167 and 773.621 ETB more than those with only mild injuries. This finding is similar to those found in studies done in developed countries where mild level of injury costs lower than moderate and severe levels of THI [7, 12, 26, 27]. This is due to moderate and severe levels of injuries requiring additional medical procedures, and more laboratory and treatment services than mild injuries.

The cost of THI increased among patients who had long lengths of stay, and lower costs among those who used medical services at other health facilities prior to the ED. Patients aged 31–45 years incurred the lowest cost compared to patients aged 5 to 14 years old.

Our study was limited to a single hospital data and cost of THI care at emergency department. In addition to this, subsequent checkups and follow-up examinations were not included. Considering this, these data indicate that the cost of THI per patient is high, and the increase in the problem will negatively impact the access to quality healthcare and household’s expenses. Therefore, it is suggested that an effort has to be made to reduce incidence of THI. To do so, THI prevention should be recognized as a priority activity in the health policy strategy.

## Conclusion

The cost of THI is relatively high. Most of the expenses are for direct non-medical costs, like transportation. Use of health services before coming to the ED was significantly associated with lower costs of THI. Whereas, costs were significantly increased by longer lengths of stay, higher level of injury, and younger age.

## Data Availability

The data underlying this findings could be accessed a reasonable request from the corresponding author Simegnew Handebo at hsimegnew@yahoo.com.

## Abbreviations

AOR: Adjusted Odds Ratio
CI: Confidence Interval
ED: Emergency department
ETB: Ethiopian Birr
GCS: Glasgow Coma Scale
SD: Standard Deviation
THI: Traumatic Head Injury
WHO: World Health Organization.
